# Human papillomavirus infection in Rwanda at the moment of implementation of a national HPV vaccination programme

**DOI:** 10.1186/s12879-016-1539-6

**Published:** 2016-05-24

**Authors:** Fidele Ngabo, Silvia Franceschi, Iacopo Baussano, M. Chantal Umulisa, Peter J. F. Snijders, Anne M. Uyterlinde, Fulvio Lazzarato, Vanessa Tenet, Maurice Gatera, Agnes Binagwaho, Gary M. Clifford

**Affiliations:** Ministry of Health of Rwanda, Kigali, Rwanda; Université Libre de Bruxelles, Ecole de Santé Publique, Brussels, Belgium; International Agency for Research on Cancer, 150 cours Albert Thomas, 69372 Lyon Cedex 08, France; Department of Pathology, VU University Medical Center, De Boelelaan 1117, 1081 HV Amsterdam, The Netherlands; Unit of Cancer Epidemiology, Department of Medical Sciences, University of Turin, Turin, Italy; Harvard Medical School, Boston, MA USA; Geisel School of Medicine, Dartmouth, Hanover USA

**Keywords:** Human papillomavirus, Human immunodeficiency virus, Prevalence, Cervical cancer, Rwanda

## Abstract

**Background:**

Cervical cancer is the most common female cancer in Rwanda that, in 2011, became the first African country to implement a national vaccination programme against human papillomavirus (HPV).

**Methods:**

To provide a robust baseline for future evaluations of vaccine effectiveness, cervical cell specimens were obtained from 2508 women aged 18–69 years from the general population in Kigali, Rwanda, during 2013/14. 20 % of women were HIV-positive. Samples were used for liquid-based cytology and HPV testing (44 types) with GP5+/6+ PCR.

**Results:**

HPV prevalence was 34 %, being highest (54 %) in women ≤19 years and decreasing to 20 % at age ≥50. Prevalence of high risk (HR) HPV and cytological abnormalities was 22 and 11 % respectively (including 2 % with high-grade squamous intraepithelial lesions, HSIL) decreasing with age. Age-standardised prevalence of HR HPV was 22 % (or 19 % among HIV-negative women), and HPV16 was the most common type. Prevalence of HPV and cytological abnormalities were significantly higher in HIV-positive than HIV-negative women, and the difference increased with age. Other significant risk factors for HPV positivity in multivariate analyses were high lifetime number of sexual partners, receiving cash for sex, and being a farmer. 40 % of women with HSIL were infected with HPV16/18 and there was no significant difference between HIV-positive and HIV-negative women.

**Conclusions:**

This study confirms Rwanda to be a setting of high prevalence of HPV and cervical disease that is worsened by HIV. These data will serve as a robust baseline for future evaluations of HPV vaccine programme effectiveness.

## Background

Cervical cancer represents by far the most common cancer among females in Rwanda, and has a high age-standardised incidence rate of approximately 42 cases per 100,000 women per year [1] that is typical of many sub-Saharan African countries. In response to this high burden, Rwanda became the first African country to initiate a national vaccination programme against human papillomavirus (HPV), the necessary cause of cervical cancer. In 2011, over 92,000 girls in primary school grade six (approximately 12 years old) were vaccinated with quadrivalent (HPV16/18/6/11) HPV vaccine. The three-dose vaccination coverage was estimated at 93 % in the target population [2]. During 2012 and 2013, a catch-up vaccination targeted girls also in secondary school grade three (approximately 15 years old) whereas from 2014, vaccination has reverted to targeting primary school grade six only.

However, the impact of HPV vaccination on cervical cancer will likely not be observed for 20 years at least, so earlier evidence of vaccine programme effectiveness is important to encourage planners in Rwanda and other African countries to sustain their programmes. To this end, the Rwandan Ministry of Health, in collaboration with the International Agency for Research on Cancer (IARC), initiated a series of studies to evaluate the early impact of HPV vaccination on HPV prevalence.

A pre-vaccination study of the prevalence of, and risk factors for, HPV infection, was initiated in 2011 according to the standard protocol of the IARC HPV prevalence surveys [3]. Cervicovaginal samples were collected from women aged 18–69 years living in the capital, Kigali, with a particular effort to oversample women ≤25 years of age in whom the impact of vaccination on HPV prevalence should be seen the earliest. The influence of HIV positivity, which is known to be an important determinant of HPV prevalence and may affect the future effectiveness of HPV vaccination, was also evaluated.

## Methods

### Population

Between July 2013 and May 2014, a survey was conducted by the Ministry of Health of Rwanda in collaboration with the IARC, Lyon, France. The study aim was to enrol 2500 women from the general population using an age-stratified approach, namely 1000 women aged below 25 years old, 500 women aged 25 to 29 years old, 200 women in each 5-year age group between 30–34 and 45–49 years, and 200 women aged ≥50 years. All mentally and physically competent women were eligible for the study, regardless of their marital status, with exception of those who were known to be pregnant.

All participants were residents of Nyarugenge District, Kigali and were recruited in two ways: 1) A population-based invitation of women aged 18–69 years residing in pre-defined areas of Nyarugenge District was made through community workers and community meetings. Women were invited to attend the Reproductive Health Department of the district hospital (Muhima). Participation rates are difficult to estimate due to the uncertainty on the number of women who had been reached by the information campaign. 2) An additional opportunistic approach was used to recruit women aged ≤29 years who were spontaneously attending Muhima hospital or eight other health centers in the Nyarugenge District (Muhima, Butamwa, Rugarama, Mwendo, Corum, Gitega, Kabusunzu, Biryogo). Principal reasons for consultation were family planning, childhood vaccination, post-natal care services and HIV-related services. Study procedures were undertaken in the centre where the woman was consulting and very few refusals were recorded.

Following signature of an informed consent form, a structured risk questionnaire was administered to all study participants, and data transferred electronically to IARC via a specifically designed on-line webpage/application.

A cytobrush (Cervex-Brush, Rovers Medical Devices B.V., The Netherlands) was used for the collection of exfoliated cervical cells from the endocervix and ectocervix for all women. The brush containing cellular material was placed in a vial containing PreservCyt medium (Cytyc, Boxbourough, MA, USA) and stored at +4C until shipment.

Women aged ≥30 years old also underwent screening by visual inspection with acetic acid (VIA). Women with visible abnormalities of the cervix, and/or who were positive for high risk (HR) HPV infection (for which results became available at a later date, see below) were to be recalled for a second VIA and treatment according to national protocols (including cryotherapy, thermo coagulation or loop electrosurgical excision of the cervix transition zone). Biopsies were to be taken from all HPV-positive women, either VIA-directed to a visible lesion or randomly from 12 o’clock of the transformation zone in the absence of a visible lesion, prior to any treatment. Histological confirmation of cervical tissue was performed at the Department of Pathology at the Kigali Teaching University Hospital. Histology results are the subject of ongoing quality control and, apart from invasive cervical cancer, are not reported here. Among women ≤29 years, no VIA was performed, nor were HR HPV-positive women recalled for follow-up.

### HPV testing and liquid based cytology (LBC)

HPV testing was performed on exfoliated cervical cells in the Department of Pathology at the VU University Medical Center, Amsterdam. DNA was extracted from the PreservCyt sample using magnetic beads (Macherey-Nagel) on a robotic system (Hamilton Robotics) according to the manufacturer’s instructions. Beta-Globin PCR analysis was conducted first to confirm the presence of human DNA in all specimens [4]. The presence of HPV DNA was determined by conducting a general primer GP5+/6+ − mediated PCR, which permits the detection of a broad spectrum of genital HPV types [5]. HPV positivity was assessed by hybridization of PCR products in an enzyme immunoassay with two oligoprobe cocktails that, together, detect 44 mucosal HPV types. Subsequent HPV genotyping was conducted by reverse-line blot hybridization of GP5+/6+ PCR products [6]. HPV types considered HR types for this analysis were HPV16, 18, 31, 33, 35, 39, 45, 51, 52, 56, 58, 59, and 68 [7]; possible HR types HPV26, 30, 34, 53, 66, 67, 69, 70, 73, 82, and 85; low-risk (LR) types HPV6, 11, 32, 40, 42, 43, 44, 54, 55, 57, 61, 64, 71, 72, 81, 83, 84, 86, 89, and 90.

Liquid-based cytology slides were prepared using a Thin Prep 3000 processor (Cytyc-Hologic), and were read at the Department of Pathology at the Vrije University Medical Center, Amsterdam, The Netherlands. Cytological diagnoses were reported according to CISOE-A standards [8] and were translated into Bethesda 2001 terminology system as 1) normal, 2) atypical squamous cells of undetermined significance; atypical squamous cells cannot exclude high-grade lesion; atypical glandular cells of undetermined significance; low-grade squamous intraepithelial lesions (ASCUS/ASC-H/AGUS/LSIL), 3) high-grade squamous intraepithelial lesions (HSIL), or 4) invasive cancer.

### Statistical analyses

Age-standardised HPV prevalence was computed using the world standardised population to allow comparisons of HPV prevalence with other IARC HPV surveys [9]. Adjusted prevalence ratios (PR) for HPV positivity and corresponding 95 % confidence intervals (CI) were computed using two binomial regression models with a log link, the first adjusted for age group (<24; 25–34, 35–44 and ≥45 years) only, and a second model that adjusted additionally for lifetime sexual partners (1, 2 or ≥3) and HIV status (positive, negative, unknown). Risk trends were assessed by considering categories as continuous variables. Effect modification of HIV status on the association between HPV16/18 positivity and cytology was tested using the log-likelihood ratio test.

## Results

### General female population

Of 2511 participants providing cervical cell samples, 2508 had valid HPV results and were included in the following analyses, including 1233 and 1275 recruited at Muhima Hospital and health centres, respectively. Ninety nine percent of women reported no HPV vaccination and 98 % no history of previous cervical cancer screening. Among the 2391 women with a valid result on LBC, 255 (11 %) had an abnormal cytological diagnosis, including 210 (9 %) with ASCUS/ASC-H/AGUS/LSIL and 45 (2 %) with HSIL.

Overall HPV prevalence was 34 %, being 29 % among women with normal cytology (Table 1). The prevalence of HPV was 73 and 96 % in ASCUS/ASC-H/AGUS/LSIL and HSIL, respectively. In total, 480 (19 %) women had single-type and 377 (15 %) had multiple-type infections. Crude and age-standardised prevalence of HR HPV types was 22 %. Prevalence of possible HR HPV and LR HPV was 10 and 15 % respectively. HPV16 was the most common HPV type in women with normal cytology (4 %), ASCUS/ASC-H/AGUS/LSIL (11 %), and HSIL (29 %). HPV52 and HPV35 were the next most common HR types in women with normal cytology and ASCUS/ASC-H/AGUS/LSIL, compared to HPV18 and HPV58 in HSIL. Four invasive cervical cancers were found in the study population (2 HPV16, 1 HPV33, 1 HPV negative), and two additional invasive cervical cancers among participants without a valid sample for HPV and cytology.

**Table 1:** Prevalence of human papillomavirus (HPV) types by cytological findings and overall, among 2508 women. Rwanda, 2013–2014

HPV type	Normal (*N* = 2136)		ASCUS/ASC-H/LSIL (*n* = 210)		HSIL (*n* = 45)		Total (*n* = 2508) ^a^	
	N	%	N	%	N	%	N	%
Negative	1510	70.7	56	26.7	2	4.4	1651	65.8
Positive	626	29.3	154	73.3	43	95.6	857	34.2
Multiple	250	11.7	85	40.5	23	51.1	377	15.0
High-risk								
16	88	4.1	23	11.0	13	28.9	131	5.2
18	31	1.5	13	6.2	6	13.3	52	2.1
31	35	1.6	12	5.7	4	8.9	54	2.2
33	29	1.4	4	1.9	3	6.7	36	1.4
35	48	2.2	20	9.5	4	8.9	75	3.0
39	22	1.0	10	4.8	2	4.4	37	1.5
45	36	1.7	12	5.7	4	8.9	55	2.2
51	29	1.4	14	6.7	2	4.4	47	1.9
52	50	2.3	20	9.5	3	6.7	76	3.0
56	38	1.8	13	6.2	4	8.9	59	2.4
58	38	1.8	17	8.1	9	20.0	68	2.7
59	28	1.3	6	2.9	0	0.0	36	1.4
68	11	0.5	2	1.0	2	4.4	16	0.6
Any	378	17.7	113	53.8	40	88.9	557	22.2
Possibly high-risk								
26	9	0.4	4	1.9	3	6.7	16	0.6
30	11	0.5	6	2.9	1	2.2	18	0.7
34	0	0.0	1	0.5	0	0.0	1	0.0
53	13	0.6	11	5.2	2	4.4	26	1.0
66	39	1.8	18	8.6	1	2.2	63	2.5
67	53	2.5	8	3.8	5	11.1	68	2.7
69	11	0.5	5	2.4	0	0.0	17	0.7
70	30	1.4	8	3.8	0	0.0	38	1.5
73	13	0.6	2	1.0	1	2.2	19	0.8
82	11	0.5	6	2.9	0	0.0	18	0.7
85	4	0.2	1	0.5	1	2.2	6	0.2
Any	167	7.8	60	28.6	10	22.2	248	9.9
Low risk								
6	28	1.3	10	4.8	0	0.0	40	1.6
11	15	0.7	4	1.9	0	0.0	20	0.8
Any	282	13.2	59	28.1	10	22.2	368	14.7

^a^Including 117 women with missing/inadequate cytology

**Fig. 1:**
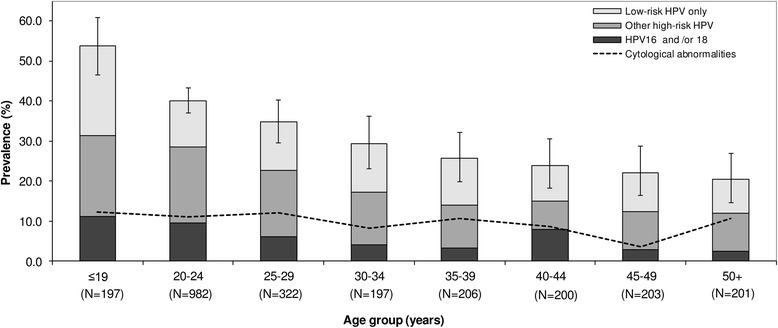
Age-specific prevalence of human papillomavirus (HPV) DNA and of cytological abnormalities among 2508 women. Rwanda, 2013–2014

Figure 1 shows the age-specific prevalence of HPV and cytological abnormalities, with HPV-positive women classified hierarchically into (1) HPV16 and/or 18, (2) other HR types and (3) LR types only. Overall HPV prevalence was highest (54 %) among women ≤19 years, and decreased with age, down to 20 % among women aged ≥50 years. Prevalence of cytological abnormalities showed no clear trend by age group.

A total of 490 (20 %) study participants self-reported to be HIV-positive, and 1414 (56 %) to be HIV-negative. An additional 604 (24 %) who reported not to know their HIV-status are hereafter considered as HIV-negative, based on similar risk factor profiles and sensitivity analyses after the exclusion of women with unknown HIV status. Eighty-four percent of HIV-positive women were receiving combined anti-retroviral therapy (cART) (data not shown). Of note, HIV-positive women represented 11 % of the study population below age 25 but 34 % in those age 45 or older.

Table 2 shows the relationship between HPV positivity and selected women’s characteristics. In the age-adjusted model, variables significantly associated with HPV positivity were age group, occupation, tobacco use, marital status, number of lifetime sexual partners, history of receiving cash for sex, the presence of anogenital warts, HIV positivity, number of pregnancies and contraceptive use in the last year. Additional adjustment for the two strongest risk factors, lifetime number of sexual partners and HIV, attenuated most PRs for HPV positivity, and the remaining significant associations included age group (PR for ≥45 vs ≤24 years = 0.45; 95 % CI: 0.37–0.55), marital status (PR for single versus married = 1.16; 95 % CI: 1.02–1.30), being a farmer (PR for farmers versus housewives = 0.78; 95 % CI: 0.66–0.92), number of lifetime sexual partners (PR for ≥3 vs 1 = 1.53; 95 % CI: 1.33–1.77), HIV positivity (PR = 1.54; 95 % CI: 1.36–1.75), number of pregnancies (PR for ≥4 vs 1 = 0.78; 95 % CI: 0.63–0.97), and use of contraceptive methods in the last year (PR for any = 0.82; 95 % CI: 0.74–0.91). Among contraceptive methods, condom did not reach statistical significance in multi-variate analysis. No significant associations with HPV positivity were seen for body mass index (5 % underweight, 7 % obese), age at menarche (median = 14 years), age at first sexual intercourse (median = 18 years), nor difference in age with partner at first sexual intercourse (median = 5 years younger) (data not shown).

**Table 2:** Prevalence ratios (PR) for human papillomavirus (HPV) positivity and corresponding 95 % confidence intervals (CIs) according to selected characteristics among 2508 women. Rwanda, 2013–2014

Characteristic	N women ^a^	HPV-positive (%)	Adjusted ^b^ PR	95 % CI	Adjusted ^c^ PR	95 % CI
Age-group (years)						
≤ 24	1179	500 (42.4)	1	-	1	-
25–34	519	170 (32.8)	0.77	0.67–0.89	0.71	0.62–0.82
35–44	406	101 (24.9)	0.59	0.49–0.70	0.52	0.44–0.63
≥ 45	404	86 (21.3)	0.50	0.41–0.61	0.45	0.37–0.55
*Chi* ^*2*^ *(1) for trend*			*p <0.001*		*p <0.001*	
Education (Years)						
Non-literate 0–5	325	122 (37.5)	1	-	1	-
Literate 0–5	640	226 (35.3)	0.86	0.73–1.03	0.97	0.82–1.14
Literate 6–10	1123	383 (34.1)	0.88	0.75–1.03	1.00	0.86–1.17
Literate ≥11	420	126 (30.0)	0.85	0.70–1.04	0.99	0.82–1.20
*Chi* ^*2*^ *(1) for trend*			*p =0.199*		*p =0.849*	
Occupation						
Housewife	964	366 (38.0)	1	-	1	-
Shopkeeper/salesman	391	154 (39.4)	1.13	0.98–1.31	1.03	0.90–1.18
Manual worker	400	132 (33.0)	0.90	0.77–1.06	0.92	0.79–1.08
Teacher/health worker/Student/Clerical staff	188	56 (29.8)	0.93	0.73–1.17	0.92	0.73–1.15
Farmer	532	134 (25.2)	0.72	0.61–0.85	0.78	0.66–0.92
Tobacco use						
Never	2276	766 (33.7)	1	-	1	-
Ever	230	91 (39.6)	1.45	1.23–1.71	1.13	0.96–1.32
Marital status						
Married or cohabiting	1734	533 (30.7)	1	-	1	-
Single	444	212 (47.8)	1.35	1.20–1.53	1.16	1.02–1.30
Separated/widowed	330	112 (33.9)	1.38	1.17–1.63	1.16	0.98–1.37
Lifetime sexual partners^d^						
1	1131	294 (26.0)	1	-	1	-
2	701	263 (37.5)	1.45	1.26–1.65	1.37	1.20–1.56
3	281	128 (45.6)	} 1.84^e^	1.62–2.10	1.53^e^	1.33–1.77
≥ 4	248	116 (46.8)				
*Chi* ^*2*^ *(1) for trend*			*p <0.001*		*p <0.001*	
Sexual activity in the last year						
Yes	2189	766 (35.0)	1	-	1	-
No	299	84 (28.1)	1.07	0.87–1.30	0.93	0.77–1.14
History of receiving cash for sex^d^						
Never	2333	769 (33.0)	1	-	1	-
Ever	154	85 (55.2)	1.59	1.37–1.84	1.14	0.98–1.33
Evidence of anogenital warts						
No	2395	814 (34.0)	1	-	1	-
Yes	113	43 (38.1)	1.35	1.07–1.72	1.09	0.88–1.35
HIV status						
Negative	1414	450 (31.8)	1	-	1	-
Unknown	604	166 (27.5)	1.01	0.87–1.18	1.02	0.87–1.18
Positive	490	241 (49.2)	1.84	1.65–2.06	1.54	1.36–1.75
Number of pregnancies						
Nulliparous	237	101 (42.6)	1.15	0.97–1.36	0.99	0.85–1.15
1	804	327 (40.7)	1	-	1	-
2-3	840	285 (33.9)	0.94	0.83–1.08	0.85	0.75–0.97
≥ 4	616	141 (22.9)	0.85	0.67–1.06	0.78	0.63–0.97
*Chi* ^*2*^ *(1) for trend*			*p =0.010*		*p =0.006*	
Contraceptive use in the last year						
No	1146	422 (36.8)	1	-	1	-
Yes	1359	432 (31.8)	0.80	0.72–0.89	0.82	0.74–0.91
Hormonal	1005	339 (33.7)	0.82	0.73–0.92	0.84	0.75–0.96
Intra Uterine Device	185	54 (29.2)	0.79	0.62–1.00	0.79	0.62–1.00
Condom	72	25 (34.7)	0.92	0.67–1.27	0.86	0.64–1.16
Other/two or more of above	97	14 (14.4)	0.48	0.29–0.78	0.45	0.26–0.78

^a^Some figures do not add up to the total due to missing values

^b^Adjusted for age as appropriate

^c^Adjusted for age, HIV status and lifetime sexual partners as appropriate

^d^three virgins excluded

^e^PR ratio is calculated for “three lifetime sexual partners or more”

**Fig. 2:**
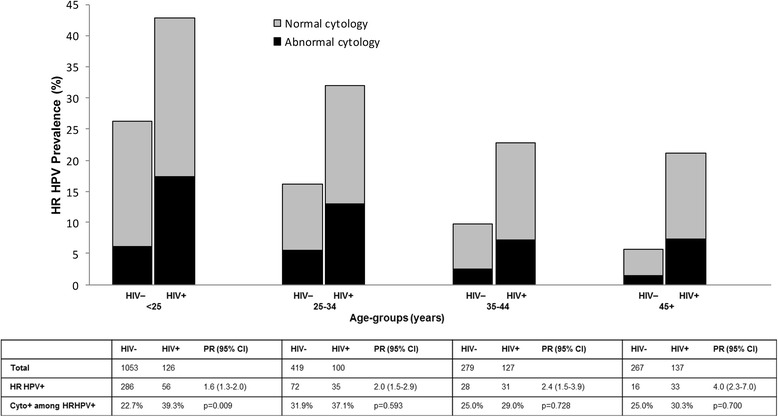
Age-specific prevalence of HR HPV infection, and of cytological abnormalities among HR HPV-positive women, stratified by HIV status among 2508 women. Rwanda, 2013–2014

In addition to total HPV prevalence, HR HPV prevalence was also significantly higher among HIV-positive (32 %) than in HIV-negative (20 %) women. Figure 2 shows prevalence of HR HPV infection, and of cytological abnormalities among HR HPV-positive women, stratified by age group and HIV positivity. PRs for HR HPV prevalence by HIV-positivity increased with increasing age from 1.6 (95 % CI: 1.3–2.0) in women below age 25 up to 4.0 (95 % CI: 2.3–7.0) in women aged 45 or older (Fig. 2). The percentage of HR HPV-positive that had cytological abnormalities was significantly higher in HIV-positive than HIV-negative women below age 25 (PR = 1.7, 95 % CI: 1.2–2.6) but similar in older age groups.

The proportion of women who were positive for HPV16/18 is shown in Table 3, by cytological diagnosis and HIV status. Overall, the proportion of HPV16/18-positive women increased from 5.5 % in normal cytology up to 40 % in HSIL with no statistically significant heterogeneity by HIV status (p for heterogeneity = 0.33) (Table 3).

**Table 3:** Positivity for human papillomavirus (HPV)16/18 among 2508 women, according to cytological findings and HIV status. Rwanda, 2013–2014

Cytology	HIV negative		HIV positive		Total	
	N	HPV16/18-pos n (%)	N	HPV16/18-pos n (%)	N	HPV16/18-pos n (%)
Normal	1749	91 (5.2)	387	26 (6.7)	2136	117 (5.5)
ASCUS/ASC-H/LSIL	146	26 (17.8)	64	8 (12.5)	210	34 (16.2)
HSIL	27	11 (40.7)	18	7 (38.9)	45	18 (40.0)
	*p*-value for heterogeneity by HIV status^a^ = 0.33					

^a^log-likelihood ratio test (see methods)

Of 976 women that underwent VIA, only 5 % were classified as positive and only one of the 22 HSILs detected in this group of women was VIA-positive (data not shown).

## Discussion

We describe the epidemiology of HPV infection in the adult female population of Rwanda soon after the country successfully embarked upon a national programme of HPV vaccination. Data represent that of an almost entirely vaccine- and screening-naive population and disclose a high prevalence of HPV infection (34 %), particularly among women ≤19 years (54 %). Findings can be robustly compared with other IARC HPV surveys that were conducted according to a similar protocol. In this respect, the age-standardised HR HPV prevalence of 22 % in Rwanda (or 19 % in HIV-negative women) ranks below that of Guinea (31 %) [10] and Mongolia (25 %) [11], is similar to the prevalence detected in Nigeria (18 %) [12] and Vanuatu (19 %) [13], but remains more elevated than that found in many other areas known to be at high-risk for cervical cancer, including that in India (12 %) [14] and Colombia (10 %) [15]. The high prevalence of HSIL and cervical cancer in the study population is consistent with recent estimates of high cervical cancer incidence in Rwanda [1].

HPV16 was by far the most frequently detected type in Rwanda, increasing from 4 % of normal cytology up to 29 % of HSIL. This agrees with a meta-analysis in which the proportion of HPV16-positive HSIL in Africa was 30 %, and lower than in other world regions [16]. Vaccine types HPV16 and/or 18 accounting for 42 % of HSIL, and a relatively high importance of HPV35 and HPV58 in Rwanda are also consistent with previous African studies [16]. The importance of HPV16 and 18 increased with the severity of cytological lesions similarly in HIV-negative and HIV-positive, and we did not observe any statistically significant under-representation of HPV16/18 in HIV-positive HSIL, as has been suggested by meta-analyses [17].

HIV infection was associated with higher prevalence of HPV, confirming previous findings from Rwanda [18–20]; sub-Saharan Africa [21–25] and elsewhere [17]. This association persisted after adjustment for lifetime number of sexual partners, confirming an independent effect of HIV-infection beyond that of confounding by sexual behaviour. Indeed, lower CD4+ cell counts (a specific measure of HIV-related immunosuppression) are known to be negatively associated with the prevalence [26–29], persistence [30], and cumulative incidence [31] of HPV infection.

In agreement with our present findings, some studies in sub-Saharan African populations have reported declining HPV prevalence with age [21, 32–34], whereas others have showed HPV prevalence remaining equally high [10, 12, 35–37], or even increasing [38] in middle and old age. Even if it declined, HR HPV prevalence remained 10–15 % in women age 35 or older in Rwanda, suggesting that a large proportion of women might test positive for HPV-based screening and require diagnostic follow-up and treatment. This problem particularly applies to HIV-positive women, among whom HR HPV prevalence decreased less with age than among HIV-negative women.

Risk factors, other than HIV and age group, associated with cervical HPV infection were mostly proxies of sexual behaviour, as identified in previous HPV prevalence surveys. Lifetime number of sexual partners, being single, and history of receiving cash for sex (albeit rarely reported), were positively associated with HPV prevalence in multivariate analyses. Multiparity and recent use of contraceptives were also significantly and negatively associated with HPV positivity. Lower prevalence in farmers than other occupational groups suggest the possibility of an urban–rural gradient in the frequency of the infection. Residual confounding by sexual behaviour on the association with occupation, multiparity, and contraceptive use is difficult to rule out [39, 40].

Although only 2 % of study participants reported a prior Pap smear, the current study coincided with the initiation of screening and treatment programmes based on VIA, and/or testing for HPV DNA with careHPV (QIAGEN, Hilden, Germany) with use of VIA to triage HPV-positives for treatment [41]. A potential warning about the performance of VIA in this setting comes from the finding that, despite very high HR HPV prevalence, VIA detected abnormalities in only 5 % of women in this high-risk population, and missed the vast majority of HSIL. This mirrors findings from other IARC HPV surveys [10, 42].

This study has strengths and weaknesses. Strengths include the relatively large number of women, especially young women, and the use of high-quality HPV testing and liquid-based cytology. HPV infection is asymptomatic and self-selection of women by level of HPV risk is unlikely to have occurred. The large number of HIV-positive women in our study will allow the opportunity to estimate HPV vaccine effectiveness in this high-risk group separately but also raises the question of whether our study population is representative of the general female population in Kigali, and Rwanda as a whole. HIV-positive women are over-represented in our survey in comparison to the general population [43]. In particular, HIV-positive women (in the vast majority under cART treatment) were clearly more willing to accept screening invitations, or to be spontaneously seeking medical care, than HIV-negative women and represented approximately one third of study women age 35 or older. Had overall age-standardised HR HPV prevalence been estimated among HIV-negative women only, however, findings would have not been very different: 19 % instead of 22 %.

## Conclusions

The findings of this study confirm Rwanda to be a setting of high prevalence of HPV and cervical disease that is worsened by HIV co-infection. These data will serve as a robust baseline for evaluations of HPV vaccine programme effectiveness in future cohorts of vaccinated girls.

### Ethics approval and consent to participate

The present study had the approval of the Health Research Committee and Rwanda National Ethical Committee of the Rwandan Ministry of Health and the IARC Ethics Committee.

Informed consent was obtained from all study participants.

### Consent for publication

Not applicable.

### Availability of data and materials

The datasets supporting the conclusions of this article are included within the article.

## Abbreviations

ASCUS/ASC-H/AGUS/LSIL: atypical squamous cells of undetermined significance, atypical squamous cells cannot exclude high-grade lesion or atypical glandular cells of undetermined significance, low-grade squamous intraepithelial lesions
cART: combined anti-retroviral therapy
CI: confidence interval
HPV: human papillomavirus
HR: high-risk
HSIL: high-grade squamous intraepithelial lesions
IARC: International Agency for Research on Cancer
LR: low-risk
PR: prevalence ratio
VIA: visual inspection with acetic acid
